# Indomethacin blocks the increased conditioned rewarding effects of cocaine induced by repeated social defeat

**DOI:** 10.1371/journal.pone.0209291

**Published:** 2018-12-17

**Authors:** Carmen Ferrer-Pérez, Tamara Escrivá Martinez, Sandra Montagud-Romero, Raúl Ballestín, Marina D. Reguilón, José Miñarro, Marta Rodríguez-Arias

**Affiliations:** Department of Psychobiology, Faculty of Psychology, Universitat de València, Valencia, Spain; Technion Israel Institute of Technology, ISRAEL

## Abstract

It is well established that repeated social defeat stress can induce negative long-term consequences such as increased anxiety-like behavior and enhances the reinforcing effect of psychostimulants in rodents. In the current study, we evaluated how the immune system may play a role in these long-term effects of stress. A total of 148 OF1 mice were divided into different experimental groups according to stress condition (exploration or social defeat) and pre-treatment (saline, 5 or 10 mg/kg of the anti-inflammatory indomethacin) before each social defeat or exploration episode. Three weeks after the last social defeat, anxiety was evaluated using an elevated plus maze paradigm. After this test, conditioned place preference (CPP) was induced by a subthreshold dose of cocaine (1 mg/kg). Biological samples were taken four hours after the first and the fourth social defeat, 3 weeks after the last defeat episode, and after the CPP procedure. Plasma and brain tissue (prefrontal cortex, striatum and hippocampus) were used to determine the levels of the pro-inflammatory cytokine interleukin 6 (IL-6). Results showed an increase of peripheral and brain IL-6 levels after the first and fourth social defeat that was reverted three weeks later. Intraperitoneal administration of the anti-inflammatory drug indomethacin before each episode of stress prevented this enhancement of IL-6 levels and also reversed the increase in the rewarding effects of cocaine in defeated mice. Conversely, this protective effect was not observed with respect to the anxiogenic consequences of social stress. Our results confirm the hypothesis of a modulatory proinflammatory contribution to stress-induced vulnerability to drug abuse disorders and highlight anti-inflammatory interventions as a potential therapeutic tool to treat stress-related addiction disorders.

## Introduction

Scientific evidence suggests that alterations of inflammatory parameters are linked to a vulnerability to mental illnesses [1], including depression [2, 3, 4], bipolar disorder [5, 6, 7], schizophrenia [8, 9, 10] and autism [11]. In addition, substance use disorders (SUD) are assumed to be related to changes in the immune system activity [12, 13]. Addiction can be considered a multifactorial mental disorder caused by an interaction between biological and environmental factors [14, 15, 16]. Although the exact mechanisms of the genesis of addiction have not been completely unraveled, a growing body of evidence relates alterations of the immune response to a possible cause of vulnerability to SUD [17, 18]. Neuroinflammation could help to explain some of the effects of drugs, including toxicity, deleterious cognitive effects [17, 19, 20, 21] and reward modulation, particularly taking into account that the immune signal significantly modulates the mesolimbic dopamine system [18, 22].

Both clinical and preclinical studies have shown that psychostimulants such as cocaine or methamphetamine activate the central and peripheral components of the innate immune system [12, 23, 24, 25, 26]. Repeated consumption of psychostimulants promotes a neuroinflammatory pattern characterized by an enhanced activation of glial and microglial cells [12] and increased release of glial cytokines with potential neurotoxic effects [27]. Moreover, plasmatic cytokines levels are under consideration by some researchers as possible biomarkers for cocaine users [24, 28].

Another cornerstone of our understanding of drug addiction is stress, with emotional stressors representing the main source of stress in humans [29]. Life-threatening situations induce a physiological response to stress which is adaptive and crucial for survival, and a failure to end that response may induce deleterious effects [30, 31]. In pre-clinical research, social defeat in an agonistic encounter is a rodent model with ecological validity that mimics real-life situations of social stress [32, 33, 34]. It has repeatedly been reported that different models of social defeat stress enhance the unconditioned and conditioned rewarding response to psychostimulant drugs and precipitate the reinstatement of drug seeking in the self-administration and conditioned place preference (CPP) paradigms [29, 34, 35, 36]. Alterations in the corticotrophin-releasing factor (CRF) neurotransmission system [35, 37, 38], epigenetic forms of plasticity [39] or inflammatory processes appear to be central to stress-induced vulnerability [4, 40, 41].

Recent basic research shows that stress induces activation of the immune system, thereby promoting the stimulation of microglia and leukocytes, altering the levels of peripheral and brain cytokines and leading to monocytes trafficking into the brain [40, 42]. Moreover, pro-inflammatory markers alter the permeability of the blood brain barrier (BBB) [43], which is also affected by social stress procedures [44]. When the BBB is compromised, peripheral immune cells can penetrate the central nervous system (CNS), thus causing or enhancing existing neuro-inflammation [45]. In this way, inflammatory processes are being posited as a link between stress and disease, by altering behavioral and neuroendocrine functions and leading to the vulnerability and enhanced sensibility to drugs that is reported after social stress procedures.

In response to this link between immune response, vulnerability to addiction and stress, some researchers have employed anti-inflammatory agents such as non-steroidal anti-inflammatory drugs (NSAIDs) as a therapeutic approach. For instance, the inflammatory potential of ethanol is well established [18, 20, 21, 46, 47]; it produces an inflammatory response via activation of microglia and astrocytes, contributing to neurodegeneration and the deleterious effects reported in alcoholics [48, 49] and in animal studies [20, 21, 50]. Pascual and coworkers [46] found that administration of indomethacin prior to ethanol binge-drinking prevented ethanol-induced brain damage in adolescent rats, as it blocked neural cell death and also attenuated short and long-term detrimental effects on cognitive and motor processes. Other researchers found that anti-inflammatory treatments also reverse cognitive impairments induced by social stress in animal models [42, 51].

Based on the above-mentioned observations, we hypothesize that the long-term sensitization to the rewarding properties of cocaine, constantly reported in experimental animals after social defeat stress, is mediated by a pro-inflammatory state. Likewise, we hypothesize that this inflammatory mechanism is also underlying the long-term anxiety-like behavior displayed by social defeated animals. In order to test these hypotheses, we first determined whether there is an increase in peripheral and brain inflammatory response after social stress by measuring plasmatic and brain levels of interleukin 6 (IL-6). We then determined if anti-inflammatory treatment with indomethacin before each stress episode blocks the inflammatory response induced by social stress, and therefore the enhanced cocaine response and anxiety-like behavior expected after social stress experiences.

## Material and methods

### Animals

A total number of 148 OF1 adult mice (Charles River, France) were used in this study. The animals were housed as previously described in detail [35] in groups of four in plastic cages (27 × 27 × 14 cm) during the entire experimental procedure. To reduce their stress levels in response to experimental manipulations, mice were handled for 5 minutes per day on each of the 3 days prior to initiation of social defeat experiences. Aggressive opponents were individually housed in plastic cages (21 × 32 × 20 cm) for a month prior to initiation of the experiments in order to heighten aggression [52]. All mice were housed under the following conditions: constant temperature; a reversed light schedule (white light on 8:00–20:00 hours); and food and water available *ad libitum*, except during behavioral tests. The experimental protocol has been approved by an Institutional Review Committee for the use of animal subjects (Comité d'Ètica d’Experimentació i Benestar Animal, number A1426847710979). Procedures involving mice and their care were conducted according to national, regional and local laws and regulations, which are in compliance with the Directive 2010/63/EU. All efforts were made to minimize animal suffering and to reduce the number of animals used.

### Drugs

The anti-inflammatory indomethacin (Sigma-Aldrich, Spain) was dissolved in 5% DMSO (dimethyl sulfoxide) and injected intraperitoneally (i.p.) at a dose of 5 or 10 mg/kg 30 minutes before each social defeat. For CPP, a dose of 1 mg/kg of cocaine hydrochloride (Alcaliber laboratory, Spain) was employed. This dose of cocaine was selected on the basis of previous CPP studies showing 1 mg/kg to be a sub-threshold dose [53, 54, 55]. All the treatments were adjusted to a volume of 0.01 ml/g of weight. Control groups were injected with physiological saline (NaCl 0.9%), which was also used to dissolve the drugs.

### Experimental design

The experimental design is depicted in Table 1. We studied whether social stress could induce an inflammatory response and if the anti-inflammatory indomethacin could modulate the effects of social defeat on the conditioned rewarding effects of cocaine (1 mg/kg). Mice received physiological saline, or a 5 or 10 mg/kg dose of indomethacin prior to each social defeat (RSD) (RSD-SAL, RSD-INDO5, RSD-INDO10) or exploration (EXP) (EXP-SAL, EXP-INDO10). Subsequently, 19 days after the last social defeat, anxiety was evaluated in the elevated plus maze (EPM) test. One day afterwards, the CPP procedure was initiated.

**Table 1:** Experimental design.

	RSD / Exploration						
	1st	2nd	3rd	4th			
**1st set of mice**	SAL/ INDO5 / INDO 10	SAL/ INDO5 / INDO 10	SAL/ INDO5 / INDO 10	SAL/ INDO5 / INDO 10	EPM	1 mg/kg cocaine CPP	Brain extraction and Blood samples
**2nd set of mice**	Brain extraction and Blood samples			Brain extraction and Blood samples	Brain extraction and Blood samples		
**Experimental day**	**1**	**4**	**7**	**10**	**29**	**31–40**	**60–70**

Biological samples were taken four hours after the first and fourth social defeats, 3 weeks after the last social defeat, and following the CPP procedure. In the case of the control group (EXP), samples were also taken four hours after the first exploration session and after the CPP procedure.

### Apparatus and procedures

#### Procedure of social defeat

The social defeat protocol performed in this study was validated and described in detail in previous research papers from our research group [35, 36, 44]. Each of the social defeat episodes consisted of three phases, each of which began by introducing the “intruder” (the experimental animal) into the home cage of the “resident” (the aggressive opponent) for 10 minutes [56]. During this initial phase, the intruder was protected from attack, but the wire mesh walls of the cage allowed for social interactions and species-typical threats from the male aggressive resident, thus leading to instigation and provocation [57]. The wire mesh was then removed from the cage to allow confrontation between the two animals for a 5-minute period. In the third phase, the wire mesh was returned to the cage to separate the two animals once again for another 10 minutes to allow for social threats by the resident. Intruder mice were exposed to a different aggressor during each episode of social defeat. The criterion used to define an animal as defeated was the adoption of a specific posture signifying defeat, characterized by an upright submissive position, limp forepaws, upwardly angled head, and retracted ears [52]. In order to minimize the physical wounding during social defeats, the 5-minute direct encounters were finished earlier in the intruder displayed submissive supine posture for more than 8 seconds or if it was bitten by the aggressor more than 12 times. All agonistic encounters were videotaped to confirm social defeat.

#### Conditioned place preference-CPP

Place conditioning consisted of three phases and took place during the dark cycle [58], following an unbiased procedure in initial spontaneous preference terms as previously described in detail [35, 59]. For place conditioning, twelve identical Plexiglas boxes with black and white equal-sized compartments (30.7 × 31.5 × 34.5 cm) separated by a gray central area (13.8 × 31.5 × 34.5 cm) were used. The CPP protocol consists of three phases, the first one being the pre-conditioning (Pre-C). In Pre-C phase, mice were given access to both compartments of the CPP box for 15 min (900 s) for 3 consecutive days. For the evaluation of the initial/natural preference, we use the data for the time spent by the animal in each compartment registered during the third (last) day of the pretest. Animals showing a strong, unconditioned aversion (less than 33% of the session time, i.e., 300 s) or preference (more than 67%, i.e., 600 s) for any compartment were excluded for the experiment. After this initial analysis of the natural preferences, a CPP box compartment (black or white) was chosen to be paired with the drug and the other one with the vehicle, taking into account that, in each group, half the animals received the treatment in the most preferred compartment and the other half in the least preferred one. Additionally, we statistically confirmed that there were no significant differences between the time spent in the drug-paired and the saline-paired compartments to avoid any preference bias before conditioning.

In the second phase (conditioning), animals underwent two pairings per day. First, they received an injection of physiological saline before being confined to the vehicle-paired compartment for 30 minutes. After a 4-hour interval, they received cocaine immediately before being confined to the drug-paired compartment for 30 minutes. In the third phase or post-conditioning (Post-C), the time spent by the untreated mice in each compartment during a 15-minute observation period was recorded. The difference in seconds between the time spent in the drug-paired compartment in the Post-C test and that spent in the Pre-C test is a measure of the degree of conditioning induced by the drug. If this difference is positive, then the drug is considered to have induced a preference for the drug-paired compartment, whereas the opposite indicates the induction of an aversion.

All groups in which a preference for the drug-paired compartment was established underwent an extinction session every 72 hours, which consisted of placing the mice in the apparatus for 15 minutes. This was repeated until the time spent in the drug-paired compartment by each group was similar to that of the Pre-C.

The effects of non-contingent administration of a priming dose of cocaine were evaluated 24 hours after the confirmation of extinction. Reinstatement tests were the same as those for the Post-C (free ambulation for 15 minutes), except for the fact that mice were tested 15 minutes after administration of the drug (half of the dose used for conditioning). This procedure was repeated with progressively lower priming doses until a non-effective priming injection was determined.

#### Elevated plus maze-EPM

The elevated plus maze (EPM) test was carried out essentially following the procedure previously described by Daza-Losada and coworkers [59]. The maze consisted of two open arms (30 × 5 × 0.25 cm) and two enclosed arms (30 × 5 × 15 cm), and the junction of the four arms formed a central platform (5 × 5 cm). The floor of the maze was made of black Plexiglas and the walls of the enclosed arms were made of clear Plexiglas. The open arms had a small edge (0.25 cm) to provide the animals with additional grip. The entire apparatus was elevated 45 cm above floor level. In order to facilitate adaptation, mice were transported to the dimly illuminated laboratory 1 hour prior to testing. At the beginning of each trial, subjects were placed on the central platform so that they were facing an open arm and were allowed to explore for 5 minutes. The maze was thoroughly cleaned with a damp cloth after each trial. The measurements recorded during the test period were number of entries and time and percentage of time spent in each section of the apparatus (open arms, closed arms, central platform). An arm was considered to have been visited when the animal placed all four paws on it. The time and percentage of time spent in the open arms and the number of open arm entries are generally used to characterize the anxiolytic effects of drugs. In addition, the number of closed and total entries indicates motor activity.

### Tissue sampling

To obtain blood and tissue samples, unperfused mice were sacrificed by cervical dislocation and then decapitated. Blood was collected from the neck into a Microvette CB 300 capillary tube (Sarstedt, Germany). Blood samples were kept on ice, and plasma was separated from whole blood by centrifugation (5 minutes, 5000G) and transferred to sterile 0.2 ml microcentrifugue tubes. Plasma samples were stored at -80°C until IL-6 concentration determination.

Brains were rapidly removed and the prefrontal cortex (PFC), striatum (STR) and hippocampus (HIP) were dissected following the procedure described by Heffner and coworkers [60] and kept on dry ice until storage at -80°C. Prior to IL-6 determination, brains were homogenized and prepared following the procedure described by Alfonso-Loeches and coworkers [61]. Frozen brain cortices were homogenized in 250 mg of tissue/0.5 ml of cold lysis buffer (1% NP-40, 20 mM Tris-HCl pH 8, 130 mM NaCl, 10 mM NaF, 10 μg/ml aprotinin, 10 μg/ml leupeptin, 40 mM DTT, 1 mM Na_3_VO_4_, and 10 mM PMSF). Brain homogenates were kept on ice for 30 minutes and centrifuged at maximum speed for 15 minutes, after which the supernatant was collected and protein levels were determined by the Bradford assay from ThermoFisher (Ref: 23227).

### IL-6 ELISA assay

To determine IL-6 concentration in plasma and tissues, we used a Mouse IL-6 ELISA Kit obtained from Abcam (Ref: ab100712) following the manufacturer’s instructions. To determine absorbance, we employed an iMark microplate reader (Bio-RAD) controlled by Microplate Manager 6.2 software. The optical density was read at 450nm and the final results were calculated using a standard curve following the manufacturer’s instructions, and were expressed as pg/ml for plasma, and pg/mg for tissue samples.

### Statistical analyses

For the CPP data, the time spent in the drug-paired compartment during Pre-C and Post-C tests was analyzed with a mixed three-way ANOVA, with two between-subjects variables—Pre-treatment, with three levels (Saline, Indomethacin 5 or 10 mg/kg), and Stress, with two levels (RSD and EXP)—and a within-subjects variable—Days, with two levels (Pre-C and Post-C). For the EPM data, a two-way ANOVA, with two between-subjects variables—Pre-treatment, with two levels (Saline or Indomethacin 10), and Stress, with two levels (RSD and EXP)—was employed. In all cases, post-hoc comparisons were performed with Bonferroni tests. In addition, the groups showing CPP, extinction and reinstatement values were analyzed by a Student’s t-test.

Data concerning IL-6 concentration were analyzed using a single factor analysis ANOVA, with a between-subjects variable: Stress, with 4 levels (Exploration, first social defeat, fourth social defeat and 3 weeks after the last social defeat). For the IL-6 levels measure performed after the CPP procedure, we used an ANOVA with a between-subjects variable: Stress, with 3 levels (Exploration, RSD and RSD plus Indomethacin). Data are presented as mean ± SEM. A p-value < 0.05 was considered statistically significant. Analyses were performed using SPSS v22.

## Results

### Indomethacin blocks the increase in the conditioned rewarding effects of cocaine (1 mg/kg) induced by social defeat stress

**Fig 1:**
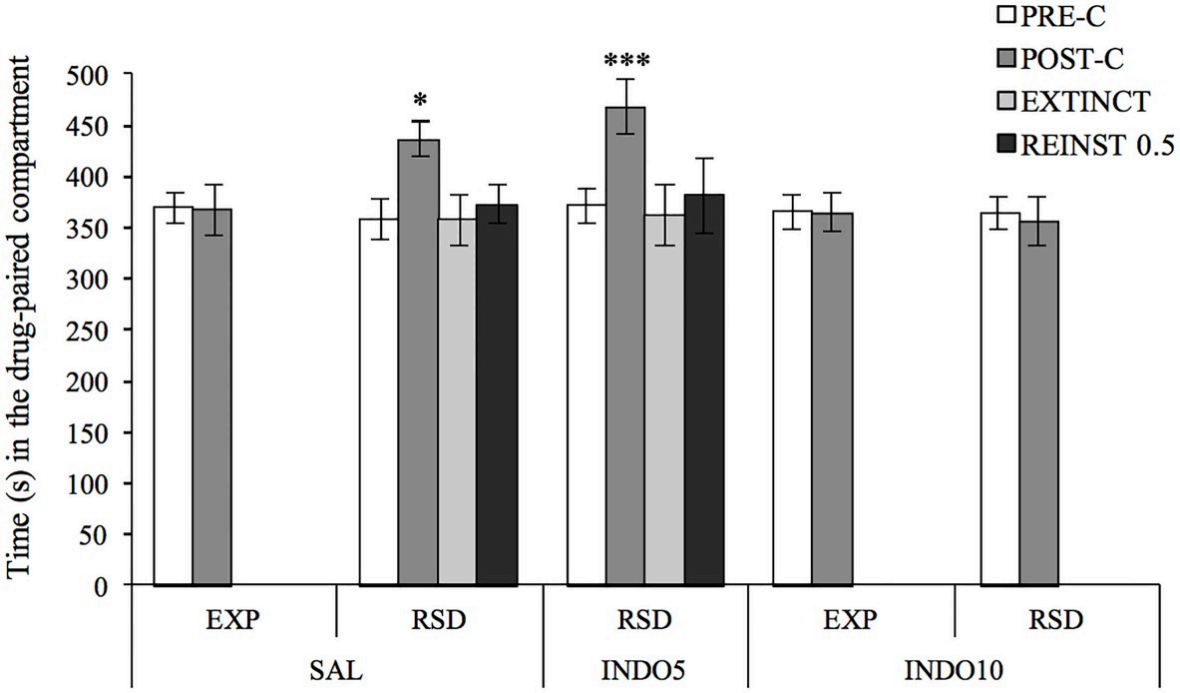
Administration of the highest indomethacin dose before each social defeat blocked acquisition of the CPP induced by 1 mg/kg of cocaine in defeated mice. Before the social stress protocol animals were randomly assigned to the following groups according to the pre-treatment they received: saline (EXP-SAL n = 13; RSD-SAL n = 14); 5 (RSD-INDO5 n = 16) or 10 mg/kg (EXP-INDO10 n = 13; RSD-INDO10 n = 17) of indomethacin. Bars represent the time (s) spent in the drug-paired compartment before conditioning sessions in the PRE-C test (white bars), after conditioning sessions in the POST-C test (dark grey bars), in the last extinction (EXTINCT) session (light gray bars), and in the reinstatement (REINST 0.5) test (black bars). Data presented as mean values ± SEM *p <0.05, ***p <0.001 significant difference in the time spent in the drug-paired compartment versus PRE-C.

ANOVA of the CPP data (Fig 1) showed a significant effect of the variable Days F(1,68) = 10.905, p <0.01 and the interactions Days x Pre-treatment F(2,68) = 4.657, p <0.05 and Days x Pre-treatment x Stress F(1,68) = 3.862, p <0.05. As expected, socially defeated animals pretreated with saline (RSD-SAL) developed CPP, since they spent more time in the drug-paired compartment in the Post-C than in the Pre-C test (p <0.05), a preference that was not observed in defeated mice treated with the highest dose of indomethacin (RSD-INDO10), in contrast with the CPP developed in defeated mice treated with the lowest dose of indomethacin (RSD-INDO5 p <0.001). Neither of the groups that developed preference for the drug-paired compartment showed reinstatement after a priming dose of 0.5 mg/kg cocaine.

#### Indomethacin fails to prevent the long-term anxiogenic effects of social defeat

The data of the EPM test are presented in Table 2. ANOVA for the time spent in the open arms F(1,59) = 18.360, p <0.001, the percentage of time spent in the open arms F(1,59) = 15.751, p <0.001, the number of entries into the open arms F(1,59) = 4.594, p <0.05, the percentage of entries into the open arms F(1,59) = 15.793, p <0.001, the time spent in the closed arms F(1,59) = 5.267, p <0.05, the number of entries into the closed arms F(1,59) = 19.641, p <0.001 and the total number of entries F(1,59) = 19.887, p <0.001 revealed a significant effect of the variable Stress. Post-hoc analyses showed that socially defeated animals spent less time, a lower percentage of time and a lower percentage of entries into the open arms (p <0.001 in all cases), while they spent more time in the closed arms (p <0.05), made more total entries (p <0.001), and more entries into the open (p <0.05) and closed arms (p <0.001) than control non-stressed animals.

**Table 2:** Administration of indomethacin before each SD fails to prevent the long-lasting anxiogenic effect of stress in the EPM.

	EXP		RSD		
	SAL	INDO10	SAL	INDO5	INDO10
**Time OA (s)**	88 ± 9	96 ± 13	60 ± 7 *	56 ± 12 *	47 ± 7 *
**% time OA**	38 ± 3	37 ± 5	25 ± 3 *	24 ± 5 *	20 ± 3 *
**Open entries**	17 ± 4	23 ± 4	33 ± 6 ***	31.3 ± 6 ***	26 ± 4 ***
**% entries OA**	39 ± 4	45 ± 2	29 ± 4 *	28 ± 5 *	26 ± 4 *
**Time in CA (s)**	146 ± 10	172 ± 18	181 ± 9 ***	186 ± 17 ***	193 ± 10 ***
**Closed entries**	28 ± 6	25 ± 2	86 ± 17 *	89 ± 21 *	80 ± 14 *
**Total entries**	45 ± 9	48 ± 5	119 ± 20 *	120 ± 24 *	105 ± 16 *

Data are presented as mean values ±S.E.M.

*p <0.05;

***p <0.001 significant difference with the exploration groups.

### Social defeat increases brain and peripheral IL-6 levels

**Fig 2:**
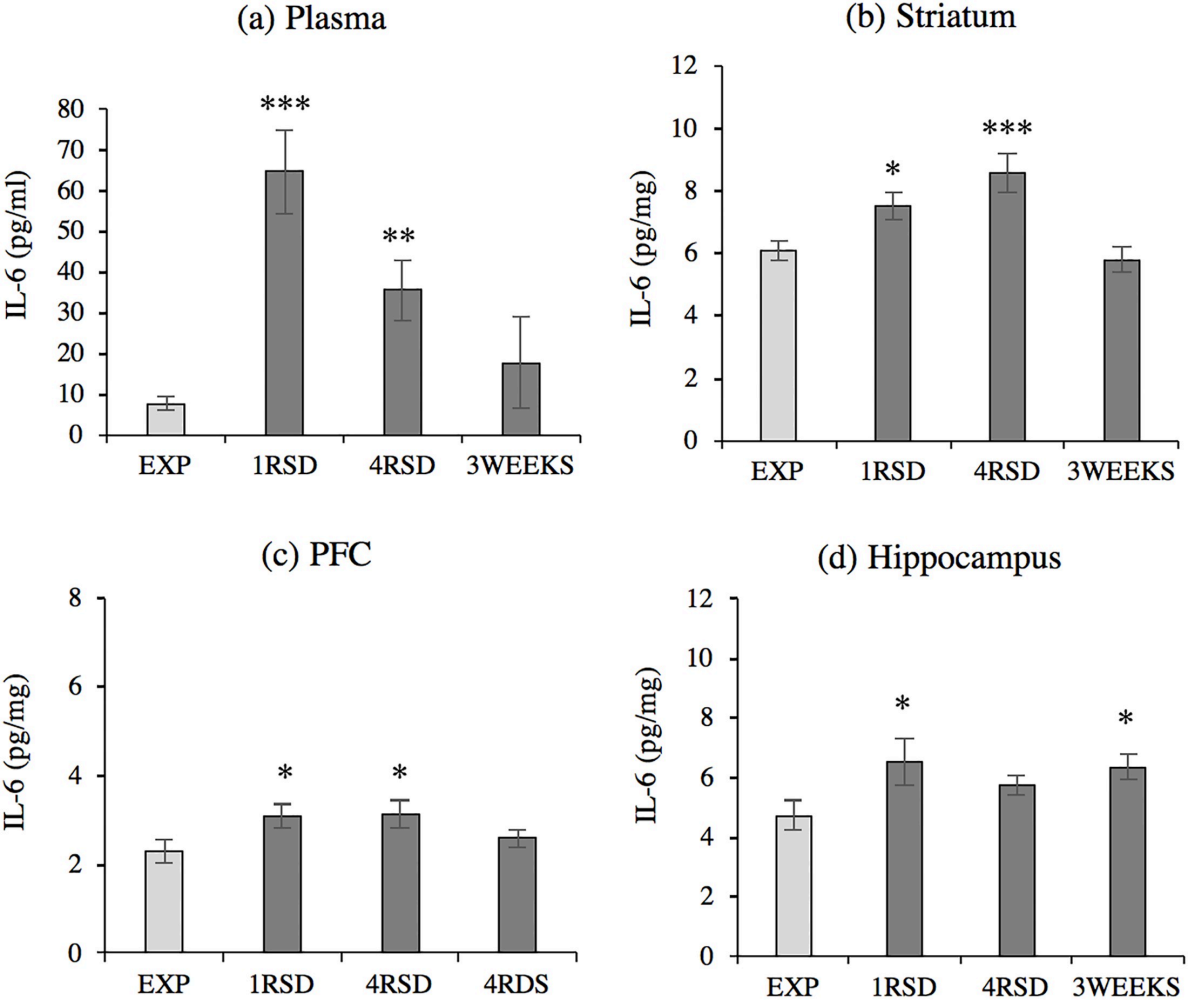
Social defeat increases IL-6 levels in the plasma, STR, PFC and hippocampus. Groups defined by stress condition and social defeat episode: (a) Plasma (EXP n = 11, 1RSD n = 11, 4RSD n = 11, 3WEEKS n = 8); (b) Striatum (EXP n = 12, 1RSD n = 12, 4RSD n = 12; 3WEEKS n = 8); (c) PFC (EXP n = 7, 1RSD n = 7, 4RSD n = 7; 3WEEKS n = 7); (d) Hippocampus (EXP n = 8, 1RSD n = 8, 4RSD n = 8; 3WEEKS n = 8). Data are presented as mean values ± SEM (pg/ml in plasma and pg/mg in brain tissue) *p <0.05; **p <0.01; ***p < 0.001 vs. exploration (EXP) group.

Regarding plasmatic circulating IL-6 levels (Fig 2a), the ANOVAF F(3,37) = 10.321, p <0.001 showed that levels were significantly increased in defeated mice after the first (p <0.001) and the fourth (p <0.01) social defeat compared with non-stressed animals. However, no differences were detected in plasma three weeks after the final exposure to stress.

Similar results were observed in the three brain areas studied. The ANOVA revealed a significant increase in IL-6 protein levels in the STR F(3,40) = 7.878, p <0.001 (Fig 2b); PFC F(3,24) = 2.991, p <0.05 (Fig 2c) and hippocampus F(3,28) = 2.891, p <0.05 (Fig 2d) after the first social defeat (p_s_ <0.05), in the STR (p <0.001) and the PFC (p <0.05) after the fourth defeat, and only in the hippocampus three weeks after the last social defeat (p <0.05).

### Indomethacin blocks the increase induced by social defeat in IL-6 levels

**Fig 3:**
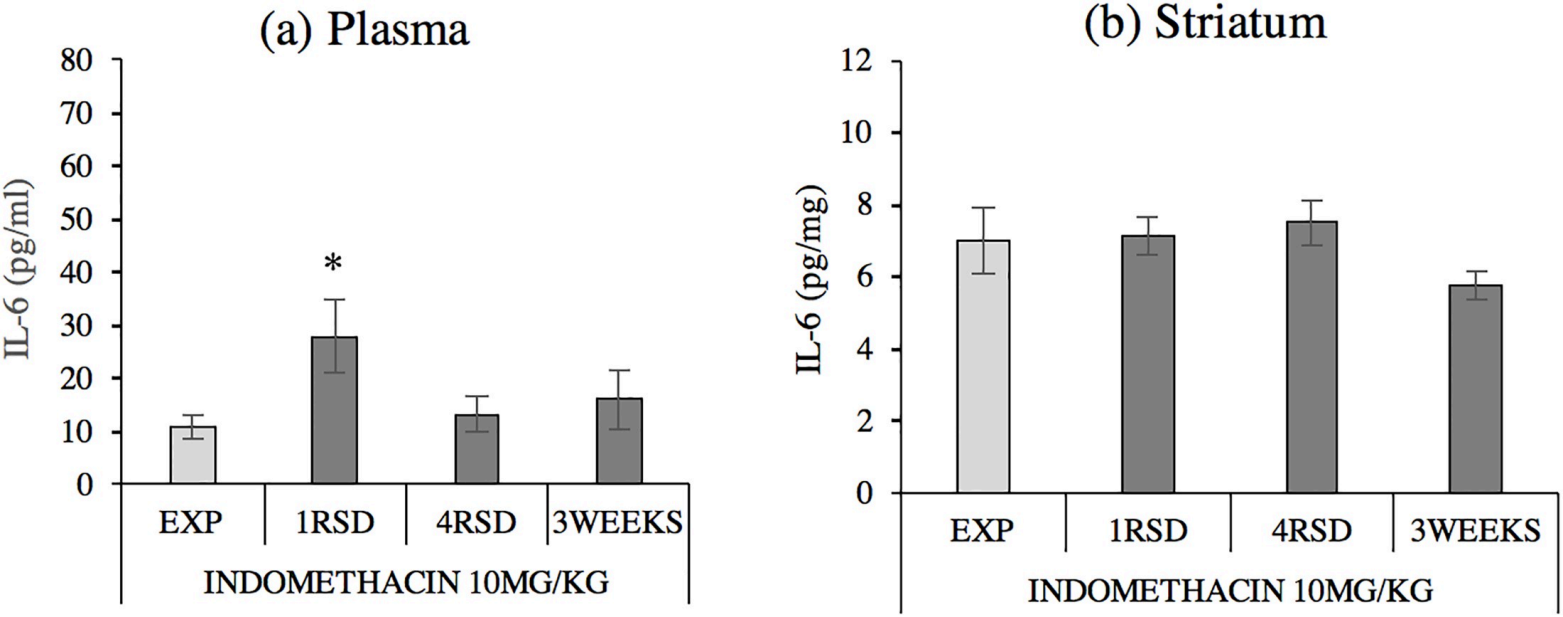
Indomethacin decreases plasma and striatum (STR) IL-6 levels in defeated mice. Groups defined by stress condition and social defeat episode, all pretreated with indomethacin 10 mg/kg: (a) Plasma (EXP n = 7, 1RSD n = 8, 4RSD n = 8, 3WEEKS = 8); (b) Striatum (EXP n = 6, 1RSD n = 7, 4RSD n = 8, 3WEEKS n = 8). Data are presented as mean values ± SEM (pg/mg) *p <0.05 vs. exploration (EXP) group.

The ANOVA F(3,27) = 2.971, p <0.05 of plasmatic IL-6 levels in animals pretreated with indomethacin before social stress (Fig 3) showed that plasmatic IL-6 protein levels were significantly increased with respect to controls in defeated mice only after the first social defeat (p <0.05). No differences were detected in the STR F(3,25) = 1.969, p >0.05.

### Increased plasma and brain IL-6 levels after cocaine-induced CPP in defeated mice

**Fig 4:**
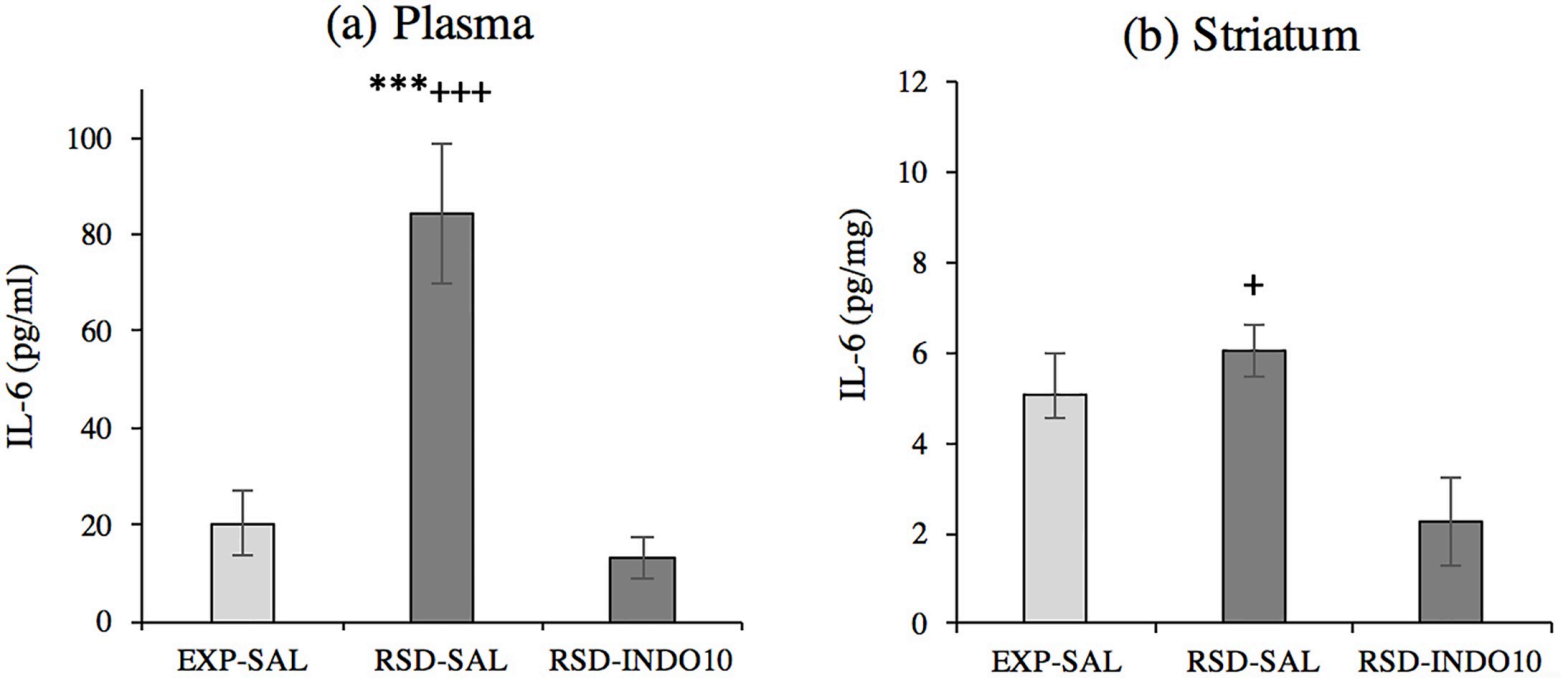
Social defeat increases plasma and striatal IL-6 levels after cocaine-induced CPP. Groups defined by stress condition and pre-treatment: (a) Plasma (EXP-SAL n = 8, RSD-SAL n = 8, RSD-INDO10 n = 8); (b) Striatum (EXP-SAL n = 6, RSD-SAL n = 6, RSD-INDO10 n = 10). Data are presented as mean values ± SEM (pg/mg); ***p < 0.001 vs. exploration (EXP) group.; +++p< 0.001; +p <0.05 vs. pretreated defeated (RSD-INDO10) group.

The ANOVA F(2,21) = 19.279, p <0.001 of plasmatic IL-6 levels after cocaine CPP (Fig 4) revealed significantly increased IL-6 protein levels in defeated mice compared to the exploration group (p <0.001). Pre-treatment with indomethacin before each social stress episode prevented this increase, as no significant differences were detected in plasmatic levels of socially defeated animals pretreated with indomethacin versus non-stressed controls, while a significant difference was observed when compared to socially defeated animals without anti-inflammatory treatment (p < 0.001).

Similar results were obtained in the STR, as ANOVA F(2,19) = 5.644, p <0.01 showed increases in IL-6 protein levels in defeated animals after CPP when compared with stressed mice previously treated with indomethacin (p <0.05).

## Discussion

In the current study, we explored the role of the immune system in the long-term effects of social stress on anxiety-like behavior and the conditioned rewarding effects of cocaine in mice. Social defeat induced a long-term increase in anxiety when evaluated with the EPM test and produced a significant increase in the conditioned reinforcing effect of cocaine in the CPP paradigm. With the aim of determining a possible role of the immune response in the genesis of these stress effects, we first verified that social defeat increased levels of the proinflammatory cytokine IL-6. Pre-treatment with the anti-inflammatory drug indomethacin before each stress episode prevented this enhancement of IL-6 levels and reversed the increase in the rewarding effects of cocaine in defeated mice. Conversely, this protective effect was not observed with respect to the anxiogenic consequences of social stress.

It has been widely demonstrated, in humans and animal models, that stressful experiences have a modulatory effect on the behavioral and physiological response to drugs [62]. In general, social stress induces a sensitization of the reward system that makes mice more sensitive to the effects of drugs [63]. In this regard, the CPP paradigm is a useful tool to study how stress can modify sensitivity to the secondary motivational properties and hedonic valence of drugs [64, 65]. In the present experiments, we observed that socially defeated animals developed CPP with a sub-threshold dose of cocaine (1 mg/kg), while this conditioned preference was not observed in animals under the exploration condition (non-stressed). These results are in agreement with previous data reported by our group for cocaine [35, 36, 44] and other substances such as alcohol or MDMA [66]. We also found that defeated animals displayed a long-term increase in anxiety-like behavior, spending less time and a lower percentage of time, and performing fewer entries and a lower percentage of entries into the open arms of the EPM than their non-stressed counterparts. This result has also been consistently replicated in the literature [66, 67, 68, 69, 70, 71].

We hypothesized that these behavioral consequences of social stress are somehow mediated by a neuroinflammatory immune response. To validate this hypothesis, we first determined if social stress could trigger an inflammatory response. We observed increased levels of the cytokine IL-6 in defeated mice four hours after social defeat episodes. Socially defeated animals displayed significantly higher plasmatic and brain (STR, PFC and hippocampus) IL-6 levels after the first and fourth social defeat when compared with exploration mice. This is not surprising, as other researchers have also reported increased levels of proinflammatory cytokines in response to social stressors [40, 68, 72]. For example, Hodes and coworkers [40] found that the higher responsiveness of the immune system to stress—characterized by higher levels of pro-inflammatory cytokines—was correlated with a higher vulnerability of mice to a stress-induced depressive-like phenotype.

However, most of these previous reports only dealt with the acute inflammatory consequences of social stress. We have focused on long term-effects in the present study by extending the timeframe of the IL-6 profile and determining its levels three weeks after the stress episode, immediately before performing the behavioral tests. Three weeks after the last social defeat encounter a significant increase in IL-6 levels was registered only in the hippocampus of socially defeated mice, which is considered a central structure in CPP establishment [73, 74]. After continuous exposure to the aggressor stimulus for 10 days, other researchers have reported an up-regulated plasmatic IL-6 levels up to 35 days after the last defeat episode [40]. We believe that these discrepancies may be a result of our shorter and intermittent social stress protocol, while the other model can be considered chronic. It should be stressed that social defeat involves physical contact during the aggressive encounter and can sometimes incur physical wounding as a consequence, which can confound the interpretation of the inflammatory measures in the brain or the blood. While some researchers did not report alterations in inflammatory markers after RSD when physical wounding is completely suppressed [75], other investigators find alterations in cytokines and in the immune response after non-physical social stress models, such as social threat exposure in juvenile mice [76] or vicarious social defeat in adults [40, 77], findings that corroborate the immune response to social stressors. We are aware of the possible interference of physical injuring in the immune response and, therefore, our protocol of RSD has been designed to minimize the physical wounding following the indications of Burke and co-workers [78].

Once we confirmed the existence of an acute immune reaction triggered by social stress episodes, we aimed to determine if the increased sensitivity to the rewarding properties of cocaine and anxiety-like behavior is somehow modulated by this pro-inflammatory response. Considering that cytokine IL-6 levels were generally similar in stressed and non-stressed mice (with the exception of the hippocampus) when they performed the anxiety and CPP tests, the different behavior of defeated mice can be explained by an initial role of the pro-inflammatory response by which long-term adaptations are promoted. For this reason, we decided to block the development of an inflammatory response by administering the anti-inflammatory indomethacin before each social stress episode. At the highest dose of indomethacin (10 mg/kg), we registered a general assuaging of the increase in IL-6 levels after the first and fourth social defeat compared with levels displayed in non-treated animals, although significantly higher levels of IL-6 in plasma continued to be measured after the first defeat when compared with the exploration group.

We also detected increased levels of IL-6 after the CPP procedure. Administration of four daily low doses of cocaine (1mg/kg) induced an enhancement of plasmatic IL-6 cytokine levels in all animals. This enhanced immune signaling was more pronounced in animals under the stress condition. Socially defeated animals presented increased IL-6 levels in plasma, with these levels proving to be statistically higher than in non-stressed animals. Again, pretreatment with indomethacin reversed this enhancement in the effect of stress, and socially defeated animals pretreated with the anti-inflammatory displayed similar IL-6 levels to the exploration group after cocaine CPP. The potential of cocaine as a xenobiotic that can activate proinflammatory central immune signaling is well documented [See revisions 17, 19]. We have found that the inflammatory potential of cocaine is exacerbated by previous stress experience, whereas an anti-inflammatory pre-treatment before stress can reverse it. It is known that previous stress experiences can sensitize peripheral and central components of the immune system [68, 69, 70]. We hypothesized that our social stress paradigm would induce long-term changes in the immune response of our experimental mice, making their immune system more reactive to insults. Indeed, indomethacin administration before each social defeat blocked the proinflammatory response induced by social stress and avoided the development of sensitization of the neuroimmune axis.

Once we had demonstrated that indomethacin was capable of reducing the release of cytokine induced by social defeat, we set out to evaluate if this decrease was related to the behavioral consequences of stress. Administration of the higher dose of indomethacin (10 mg/kg) before each social defeat completely reversed the stress-induced increase in the rewarding properties of cocaine, since defeated mice treated with this anti-inflammatory did not develop CPP induced by cocaine. One possible mechanism by which neuroinflammation can enhance the rewarding properties of cocaine is the activation of the hypothalamus hypothalamic-pituitary-adrenal (HPA) axis. It had been demonstrated that IL-6 promotes the activation the HPA axis at the hypothalamic level [79, 80], leading to the release of CRF. CRF modulates dopamine function due to its interaction within the ventral tegmental area, a key structure for drug reward effects [81, 82]. Elevated CRF levels would alter the function of the dopaminergic neurons, causing long-term neuroadaptations along this pathway [83, 84]. In this sense, increased levels of CRF in the ventral tegmental area have recently been related to increased rewarding properties of cocaine in the self-administration paradigm [85, 86].

Conversely, the anti-inflammatory treatment failed to prevent anxiety-like behavior in our socially defeated animals. While there is general agreement in the literature about the link between an enhancement of IL-6 levels and the induction of anxiety (See revision on [87]), the decrease of IL-6 levels was not effective in blocking the anxiogenic consequences of social defeat in our study. Hodes and collaborators [40] reported similar results in an experiment carried out with socially stressed mice pretreated with an IL-6 monoclonal antibody (mAbs), a pharmacological intervention that neutralizes circulating IL-6 cytokine, thereby preventing it from binding to IL-6 receptors in cell membranes. The authors found that chronic administration of IL-6 mAb prevented the development of social avoidance, which they considered a marker of susceptibility to stress consequences, while it failed to reduce anxiety-like behavior induced by stress in the EPM. One possible explanation of this discrepancy between the depressive and anxiogenic consequences of social stress may be different mechanisms for their genesis. Regarding this, Wohleb and collaborators [68, 69, 70] proposed a neuroinflammatory mechanism for the genesis of anxiety that is in line with our present results. They theorized that the pro-inflammatory profile induced by stress alters the function of vascular endothelial cells in the BBB, allowing peripheral monocytes to traffic into the brain, leading to the development of anxiety. They confirmed this hypothesis using transgenic knockdown mice for the proinflammatory interleukin-1 receptor (IL-1R1) in endothelial cells. These transgenic mice are protected from increased permeability of the inflammatory signals between brain resident microglia and peripheral blood monocytes, and, as a result, have a decreased central inflammation and did not develop anxiety-like behavior after social stress when compared with wild type mice [70]. Additionally, Wohleb and collaborators reported that this monocyte infiltration into the brain occurs in regions specifically associated with fear, anxiety and threat appraisal such as the prefrontal cortex, the hippocampus, or the amygdala while it is not registered in other regions such as the motor or somatosensory cortex, and the STR [88]. This region specificity of monocyte infiltration could explain why indomethacin failed to block the genesis of anxiety while it was effective in blocking the stress-induced increase in the rewarding properties of cocaine. As the indomethacin treatment attenuated but did not completely block the increase of peripheral IL-6 after the first RSD, we hypothesize that this increase was enough to start the mechanism that led to increases in the permeability to BBB. The decreased integrity of the BBB let monocytes traffic into the more permeable brain regions, while other regions less susceptible for this peripheral infiltration, such as the striatum (key in reward drug response), were not affected.

## Conclusions

The results of this research surpass current basic knowledge and take on a clear translational relevance, since IL-6 levels have been found to be altered in humans under conditions of social stress [83], and even more so in those with cocaine use disorders [17]. The confirmation of the contribution of inflammation to stress-induced vulnerability to mental-disorders provides new research opportunities, especially in the field of drug abuse disorders; from considering inflammatory parameters as a possible biomarker for diagnosis, to developing anti-inflammatory strategies as preventive or therapeutic interventions.

## Supporting information

S1 File CPP experimental data. (XLSX)

S2 File EPM experimental data. (XLSX)

S3 File IL-6 concentration data. (XLSX)
