# Correction: Indomethacin blocks the increased conditioned rewarding effects of cocaine induced by repeated social defeat

**DOI:** 10.1371/journal.pone.0212397

**Published:** 2019-02-11

**Authors:** Carmen Ferrer-Pérez, Tamara Escrivá-Martinez, Sandra Montagud-Romero, Raúl Ballestín, Marina D. Reguilón, José Miñarro, Marta Rodríguez-Arias

The second author’s name is spelled incorrectly. The correct name is: Tamara Escrivá-Martinez. The correct citation is: Ferrer-Pérez C, Escrivá-Martinez T, Montagud-Romero S, Ballestín R, Reguilón MD, Miñarro J, et al. (2018) Indomethacin blocks the increased conditioned rewarding effects of cocaine induced by repeated social defeat. PLoS ONE 13(12): e0209291. https://doi.org/10.1371/journal.pone.0209291

The affiliation for the second author is incorrect. Tamara Escrivá-Martinez is not affiliated with #1 but with #2 Department of Personality, Evaluation and Psychological Treatment, Faculty of Psychology, Universitat de València, Valencia, Spain.
